# Design and process optimization of a new reaction chamber for microwave synthesis of MOFs materials

**DOI:** 10.1016/j.heliyon.2024.e41304

**Published:** 2024-12-17

**Authors:** Jiadai An, Xianying Dai, Ying Liu, Kama Huang, Dengke Zhang

**Affiliations:** a School of Physics and Telecommunication Engineering, Zhoukou Normal University, Zhoukou, 466000, China; b School of Microelectronics, Xidian University, Xi'an, 710071, China; c School of Electronic Engineering, Xidian University, Xi'an, 710071, China; d College of Electronic and Information Engineering, Sichuan University, Chengdu, 610065, China; e School of Computer Science and Technology, Xidian University, Xi'an, 710071, China

**Keywords:** MOFs, Microwave synthesis, Multi-physics simulation, Orthogonal experimental

## Abstract

Based on the application demand of metal-organic framework (MOFs) materials in environmental science, energy conversion, biomedicine and other fields, its efficient synthesis method has attracted much attention. Microwave method has become one of the most competitive and potential methods because of its low cost, high efficiency and green environmental protection. However, the traditional microwave assisted synthesis of MOFs materials mostly uses microwave oven as the reaction chamber, or small-scale microwave reactor. There are many problems such as uneven heating, low microwave utilization rate and low one-time synthesis yield. In view of the above problems, the design and process optimization of a new type reaction chamber for microwave synthesis of MOFs materials were studied in this paper. A material synthesis reactor using waveguide as microwave source was designed. The structure model of the reactor was optimized by multi-physics numerical simulation, and better heating uniformity and microwave utilization were obtained. At the same time, the optimization test of key process parameters was designed by orthogonal method, and the optimal process parameter combination of this model for the synthesis of MOFs materials was obtained: microwave power is 200 W, irradiation time is 100 min, and reagent concentration is 50 mM/L. This study will promote the microwave method to achieve more efficient and uniform MOFs material synthesis, which is of great significance for further improving material yield and properties and industrial practical applications.

## Graphical abstract

A pipeline microwave reaction device was proposed in this research. Based on the numerical calculation of multiple physical fields, the geometric structure of the reaction unit is optimized, and the heating uniformity is effectively improved. Through orthogonal experiment and error analysis, the key process parameters of mof synthesis were optimized, and the optimal combination suitable for the model was obtained: microwave power of 200 W, irradiation time of 100 min, reagent concentration of 50 mM/L.

**Figure:**
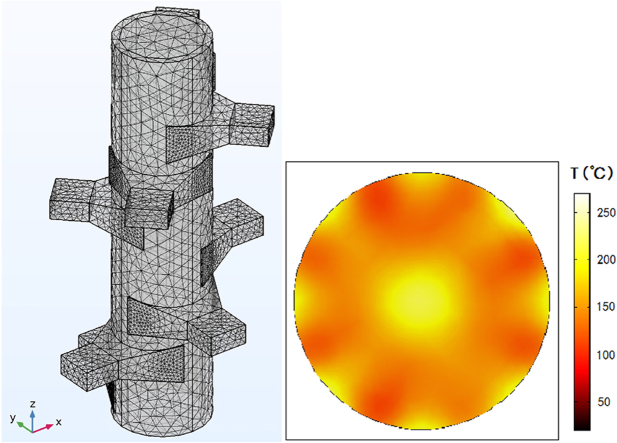

## 1 Introduction

Metal Organic Frameworks (MOFs) material has highly adjustable, large specific surface area, complex pore structure, easy to function and other properties [1,2]. As a key material in the field of the Gas storage, separation, catalysis, optoelectronics and biomedicine, it has attracted wide attention [3]. In the field of gas adsorption and separation, MOFs materials are ideal for hydrogen storage due to the high specific surface area and porosity, which can efficiently store and release hydrogen [4]. Based on the high adsorption properties of MOFs materials, carbon dioxide and methane can be efficiently captured, which is of great significance for reducing greenhouse gas emissions [5,6]. In the field of catalysis, 2D conjugated metal-organic framework materials exhibit high electrical conductivity in electrocatalysis, especially in oxygen and hydrogen precipitation, carbon dioxide reduction, oxygen reduction reactions in fuel cells, etc. [7] By combining MOF materials with some functional materials as composite materials, it can be used as electrode materials for supercapacitors to achieve long-term cyclic performance [8]. In the field of drug delivery, the nanoscale pore structure of MOFs materials makes it an ideal carrier for drug delivery system, which can control the release of drugs and improve the effect and safety of drugs [9]. At the same time, MOFs materials have and other applications with the outstanding material properties. For example, the application of MOFs materials in the field of sensors can realize sensitive detection of specific substances. The application of MOFs materials in the energy storage, such as an electro-catalyst for Al-air batteries, which having high electrochemical activity [10]. MOFs materials show outstanding potential for green ammonia synthesis and hydrogen production from ammonia decomposition, contributing to sustainable energy production [11]. Based on the huge application advantages and development potential, progressing the efficient and high-quality synthesis methods for MOFs is of great significance [12,13].

The purpose of the synthesis of MOFs is to establish the synthesis conditions, and a periodic mesh skeleton crystal structure with metal ions as the connecting points should be obtained without decomposes the organic ligand [14,15]. Microwave (MW) assisted synthesis method has been widely concerned because of its high efficiency, low energy consumption, green environmental protection and other advantages, which can achieve simple, low-cost and energy-efficient synthesis of MOFs [16]. However, there are still some problems during the microwave assisted synthesis of MOFs, such as low reproducibility, uneven heating and difficult process control, which to a large extent limit the universality of microwave assisted synthesis and restrict its large-scale industrialization [17]. In this paper, a pipeline microwave device for the MOFs synthesis is optimized and designed, and the process optimization is carried out, which effectively improved the heating efficiency, uniformity and one-time MOFs synthesis capacity, which is of great significance for the large-scale synthesis and the promotion of microwave assisted synthesis.

Knowledge of the temperature distribution of heated materials is essential when optimizing microwave assisted synthesis processes [10]. The temperature field can be determined experimentally or predicted numerically experiment. However, due to the interference of measuring devices with the electromagnetic field and the continuous nature of the process the measurement by experimental means is limited or requires high time and economic cost [18,19]. As such, numerical methods have been used to understand and optimize the microwave heating process [20]. However, we find that there is no mature numerical simulation model that can describe the microwave assisted synthesis process of MOFs. The process parameters of microwave assisted synthesis of various MOFs are only obtained through experimental tests, so there has certain limitations [21]. Many research results show that the experimental conditions will directly affect the effect of synthesis, and the reproducibility of synthesis condition is not high, which limits the microwave assisted synthesis method for large-scale popularization and developing [22,23].

The finite element method (FEM) is an efficient and high-precision numerical calculation method, which can solve some complicated mathematical and engineering problems by approximate solving of mathematical boundary value problems [24]. Based on the FEM, the numerical calculation and optimization design of a microwave synthesis reaction device and process parameters are realized in this paper. This study is based on COMSOL Mutiphysics 5.5 computing platform, which is based on the Finite Element method to achieve numerical calculation. The problems of microwave propagation, energy exchange and chemical reaction in the process of microwave synthesis of MOFs materials are transformed into the corresponding partial differential equation solving problems respectively. Based on the characteristics of microwave selective heating and penetrating heating, the coupling terms of each partial differential equation are constructed to realize the coupling between the equations of each physical field. Finally, the numerical simulation of multiple physical fields is realized. The numerical model of is carried out considering the high-frequency electromagnetic, heat transfer, mass transfer, fluid flow and other physical fields of microwave assisted synthesis process. A three-dimensional model of pipeline microwave reaction device for the MOFs synthesis is presented. Orthogonal experimental was used to design the microwave assisted synthesis process. The influence degree of each parameter is evaluated scientifically, and the optimal combination of simulation process parameters is calculated and proposed.

## 2 Calculation modeling

### 2.1 A pipeline microwave reaction device

On the basis of the traditional microwave heating reaction device structure, the microwave source and the reaction device structure are improved. Microwave is fed into the reaction region by some waveguides with a trumpet structure which are surround the reaction region, in order to improve radiation efficiency, microwave utilization and heating uniformity. The influence of pipeline radius, number of waveguides, distribution mode and other factors on heating efficiency and uniformity was studied.

**Fig. 1:**
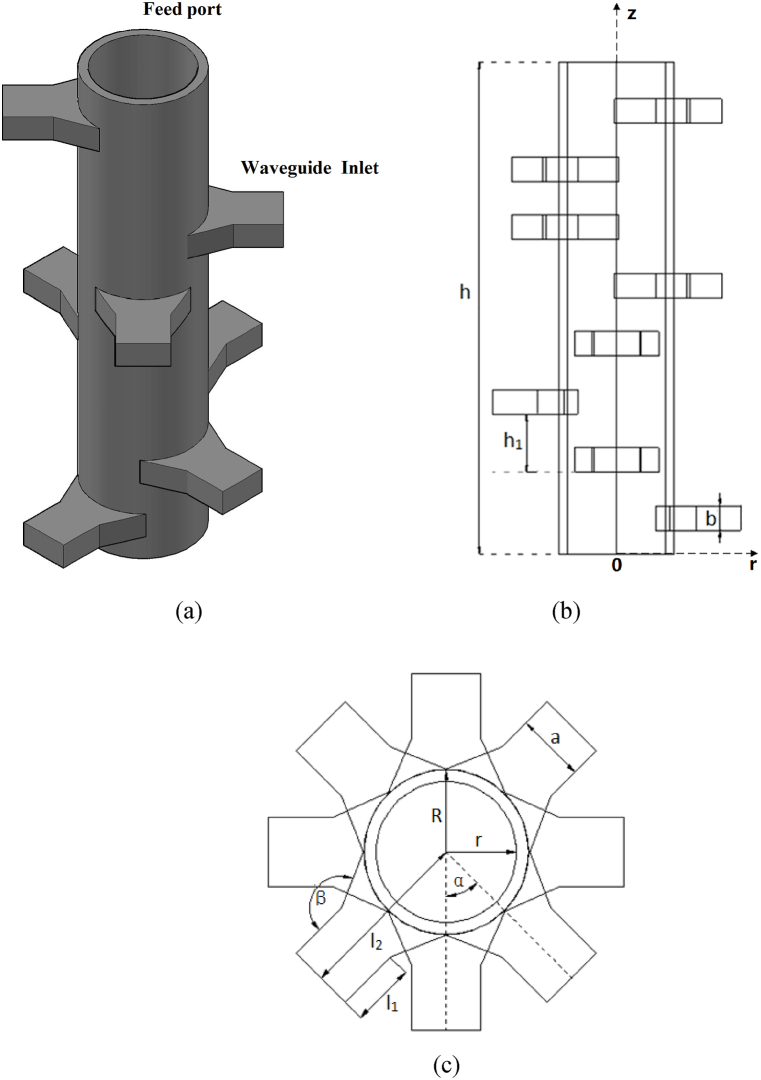
Model diagram of pipeline microwave reaction device for MOFs synthesis. (a) stereogram; (b) Side view; (c) Top view.

**Fig. 2:**
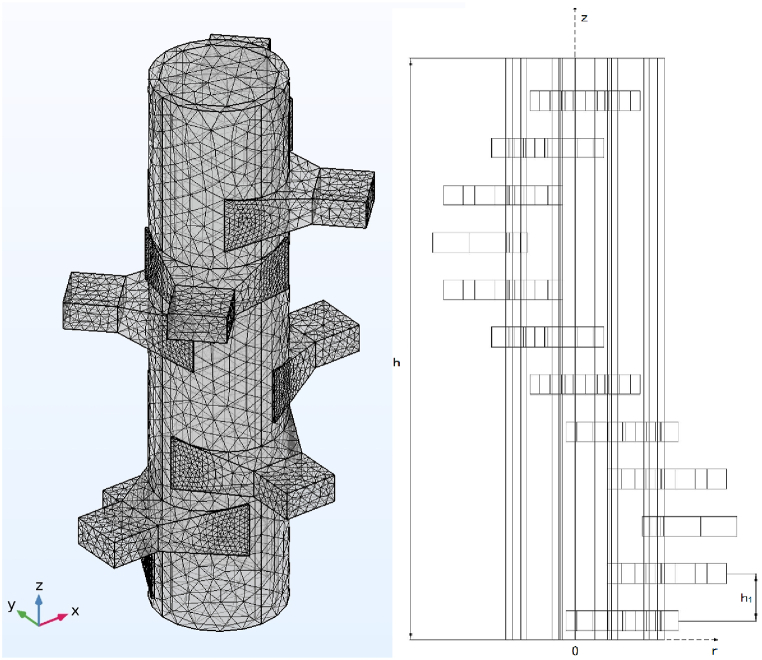
The mesh model of pipeline microwave reaction device.

As the Fig. 1. (a) shows, the structure and calculation model of the reaction device designed in this paper. The reaction device consisted of the rectangular waveguide with trumpet structure, a pipeline reaction chamber and a perfectly matched layer on the inner wall of the pipeline. Microwaves are input through the waveguide, and the reaction solution to synthesize MOFs is loaded in the pipeline reaction chamber. The dimensions of the reaction device are described in Fig. 1 (b) (c). The height of the device is h, the outer diameter of the pipeline reaction chamber is R, and the inner diameter is r. The perfect matching layer is attached to the inner wall of the pipeline, which can make the microwave fully radiate into the pipeline and prevent the harmful impurities generated by the reaction from entering the waveguide and corroding the device. The whole numerical simulation process is based on a three-dimensional model, which adopts the regional mesh refinement method to ensure the accuracy of this simulation. The mesh distribution model is shown in Fig. 2.

### 2.2 Governing equations

In this paper, a typical MOFs material HKUST-1 (Cu-BTC) was selected as the research object to optimize equipment and process parameters during simulation.

In the multi-physics coupling calculation, first of all, the chemical reaction module is used to simulate the material synthesis process, and the reaction formula is as follows:

(1) Zn(NO_3_)_2_·6H_2_O + C_8_H_6_O_4_+C_3_H_7_NO + H_2_O→C_24_H_12_O_13_Zn_4_

Then, in this model, the coupling of electromagnetic field and heat conduction is calculated, and Maxwell equations are used to calculate the electric field intensity distribution in the device:

(2) $$\begin{array}{l} \nabla \times \vec{H}=\frac{\partial \vec{D}}{\partial t}+\vec{J}\\ \nabla \times \vec{E}=-\frac{\partial \vec{B}}{\partial t}\\ \nabla \cdot \vec{D}=\rho \\ \nabla \cdot \vec{B}=0 \end{array}$$

According to Maxwell equations, the power loss per unit volume is calculated as follows:

(3) $$P_{d}=\frac{1}{2}\left(\vec{E}\cdot \frac{\partial \vec{D}}{\partial t}-\vec{D}\cdot \frac{\partial \vec{E}}{\partial t}\right)+\vec{J}\cdot \vec{E}$$

Finally according to the thermal field equation:

(4) $$\rho_{m}C_{\rho }v_{z}\frac{\partial T}{\partial t}=\nabla \cdot \left(K\nabla T\right)+P_{d}$$

The temperature field distribution in the device is obtained.

### 2.3 Orthogonal experimental model of microwave assisted synthesis processing

**Fig. 3:**
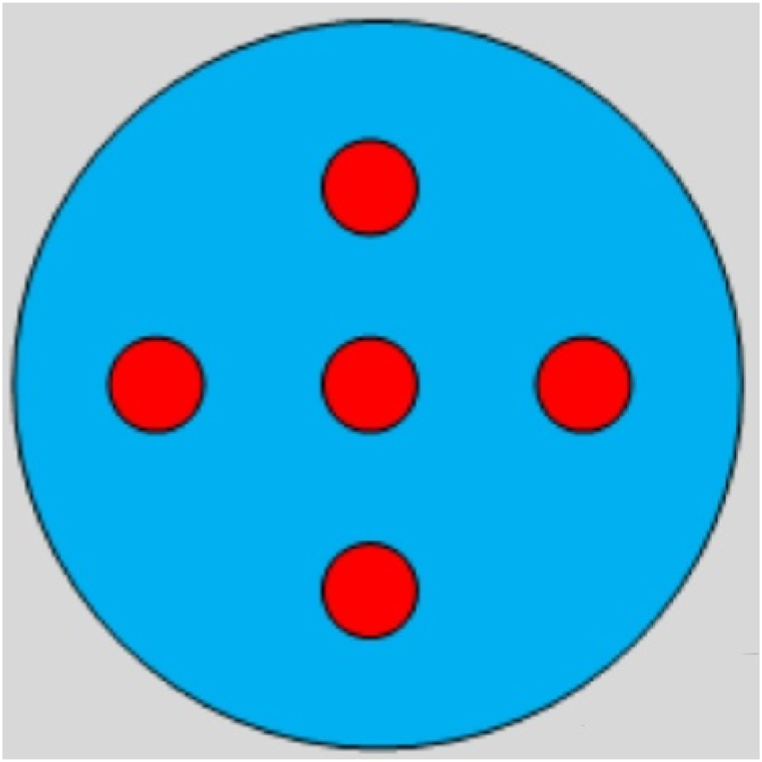
5-point diagram of the reaction chamber cross-section.

The single variable method is always used to simulate and optimize the process parameters [25]. The disadvantage of this method is that each parameter cannot be changed at the same time, and a large number of simulations are needed to achieve the results, which is likely to miss the optimal result. Orthogonal experimental is an efficient, rapid and economical scientific design method for studying multi-factor and multi-level tests [26,27]. According to the five-point principle of heating uniformity characterization, the test points were selected in the orthogonal experiment as shown in Fig. 3. And the error analysis on the multi-physics simulation results at five points of the reaction chamber cross-section to quantify the uniformity and yield of the MOFs by microwave assisted synthesis.

In the orthogonal experiment, the microwave power, irradiation time, operating pressure and reagent concentration were selected as orthogonal factors, and four levels (i.e., variables) were selected for each factor. The orthogonal experiment was designed according to the technological parameters selected in Table 1.

**Table 1:** Orthogonal factor and level.

Factors	Level				Unit
	1	2	3	4	
A Microwave power	100	200	500	1000	W
B Irradiation time	10	100	500	1200	min
C Reagent concentration	5	50	100	500	mM/L

## 3 Results and disussion

### 3.1 A pipeline microwave reaction device

#### 3.1.1 Multi-physics simulation and heating uniformity optimization

Based on the structure of the pipeline microwave heating reaction chamber, the effects of structural parameters on the thermal efficiency and thermal field distribution were simulated and analyzed. A new structure optimization model of a pipeline microwave reaction device for MOFs synthesis is proposed.

There is no uniform and strict definition standard for microwave heating uniformity. In this paper, the uniformity definition is adopted for temperature field. The coefficient of variation (COV) is used to characterize the microwave heating uniformity, which is the ratio of the data standard deviation to the mean value, expressed as follows:

(5) $$COV=\frac{\sqrt{\frac{1}{N}\sum_{i=1}^{N}{\left(T_{i}-\bar{T}\right)}^{2}}}{\bar{\Delta T}}$$

**Fig. 4:**
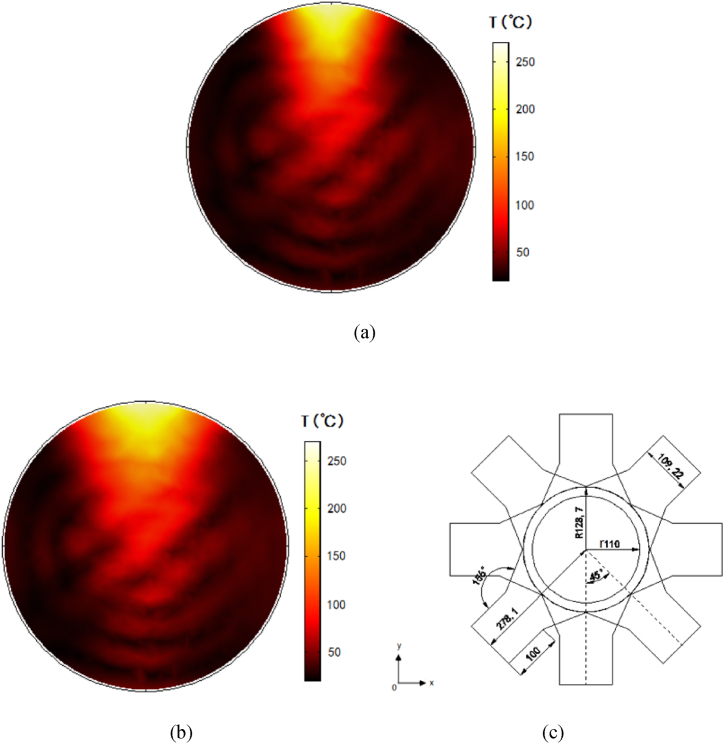
Schematic diagram of the electric field strength (a) without the trumpet structure; (b) with the trumpet structure.

In Formula (5), the denominator is the average temperature rise of the heated material, and the numerator is the standard deviation of the temperature field. N represents the number of value points of the heating cross-section, Ti represents the temperature at each point, represents the average temperature of the cross-section, and represents the average temperature rise. Since the average values of each group of temperatures are different, coefficient of variation (COV) has the advantage of eliminating the influence of measurement scale and dimension. Although there is no dimension, it is standardized according to the data mean size, which can more objectively express the degree of data dispersion. The smaller the temperature field value, the smaller the dispersion of temperature distribution, that is, the better the uniformity of microwave heating [28]. Firstly, we compare the electric field distribution in the waveguide with and without the trumpet structure. As shown in Fig. 4 (a), the electric field radiation of the waveguide without the trumpet structure generates a small range, which is mainly concentrated in the position of the port. The structure of the microwave feeder is designed as a trumpet structure. As shown in Fig. 4 (c), a flare of about 156°is used to improve the feed shape. The temperature field calculation results are shown in Fig. 4 (b). The electric field radiation range formed by the waveguide with the trumpet structure is obviously increased, and the microwave energy is more spreaded out during the reaction region, and the uniformity of microwave heating is improved.

As the trumpet structure determined, we next optimized the number of waveguides. Taking into account the model size and calculation amount, the pipe inner diameter r is temporarily set as 100 mm, the number of waveguides is n, and the rotation angle between adjacent waveguides is α. The simulation calculation values are shown in Table 2:

**Table 2:** Number and angle between distribution of waveguides.

n	1	2	3	4	5	6	8	10
α(°)	0	180	120	90	72	60	45	36

**Fig. 5:**
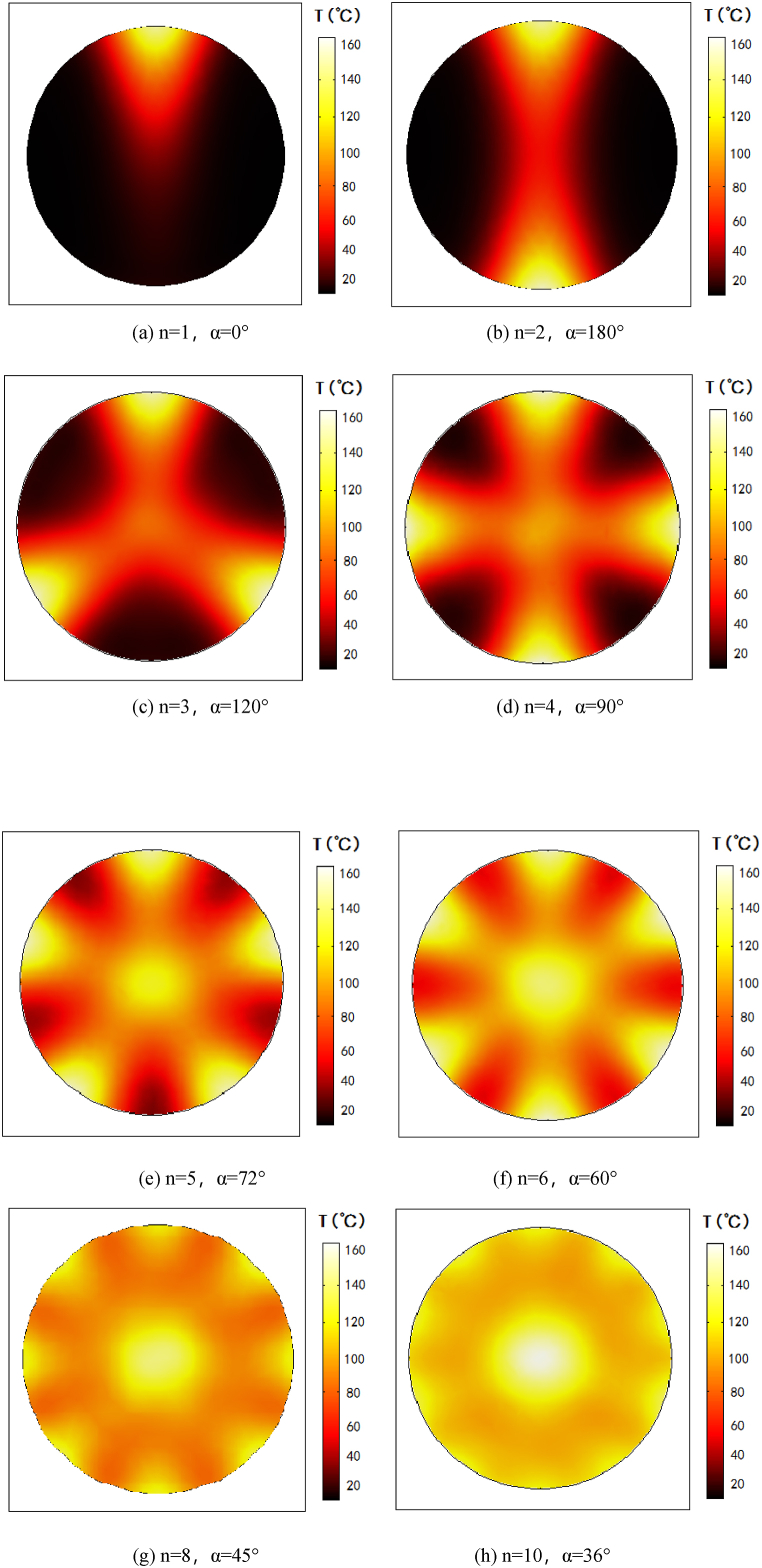
Temperature distribution cross-section under different number of waveguides.

The temperature distribution under the condition of 1∼10 waveguides and corresponding distribution angles were obtained by simulation calculation, as shown in Fig. 5.

**Fig. 6:**
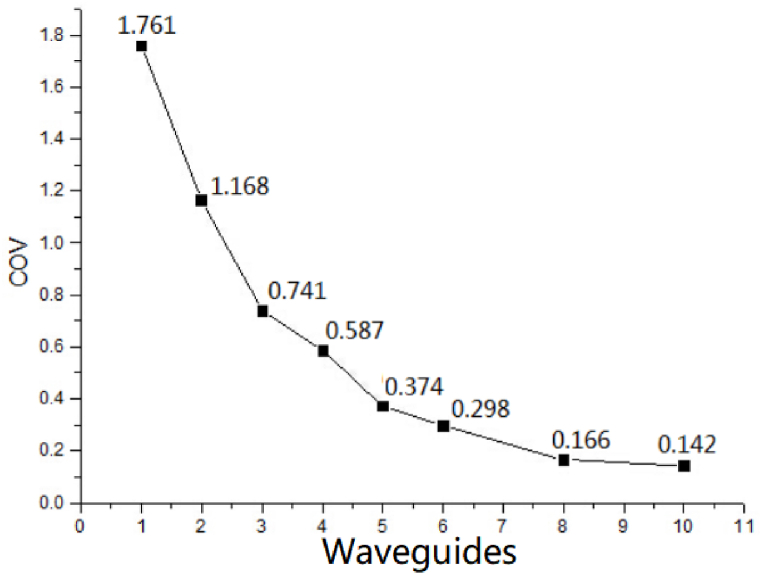
Temperature field COV curve of different number and distribution of waveguides.

As the Fig. 5 shows, when there is only one waveguide inlet, the heating region is very concentrated, and almost only the waveguide inlet area generates temperature rise. When the waveguide increased to two opposite structure, the temperature rise area is significantly larger than that of one inlet. By analogy, from (a)–(h) in Fig. 5, with the increase of waveguides, the temperature rise area becomes larger. At the same time, it can be clearly seen from that the color difference of the microwave heating area is getting smaller and smaller, which illustrated the temperature distribution is getting more and more uniform. By further using temperature field COV to compare the microwave heating uniformity under different waveguide numbers, the results are shown in Fig. 6.

The horizontal axis in Fig. 6 represents an increase in the number of microwave feeders from 1 to 10. The vertical axis represents a gradual decrease in the COV. With the increase of the number of waveguide feeders, the temperature field value decreases as a whole. The smaller the number of waveguide, the larger COV of the temperature field. The larger the number of waveguide, the smaller COV of the temperature field, indicating the better heating uniformity. As the Fig. 6 shows, the COV curve shows that the larger the number of waveguides, the smaller the temperature field COV. However, when the waveguides increase to 8 and 10, the COV values are 0.166 and 0.142, respectively, with little difference. Therefore, in order to ensure the heating uniformity, considering the microwave energy saving and the compact design of the device, the best design scheme is to select 8 waveguide feeders, which are distributed in an array with an equal Angle of 45°around the pipeline reactor chamber.

**Fig. 7:**
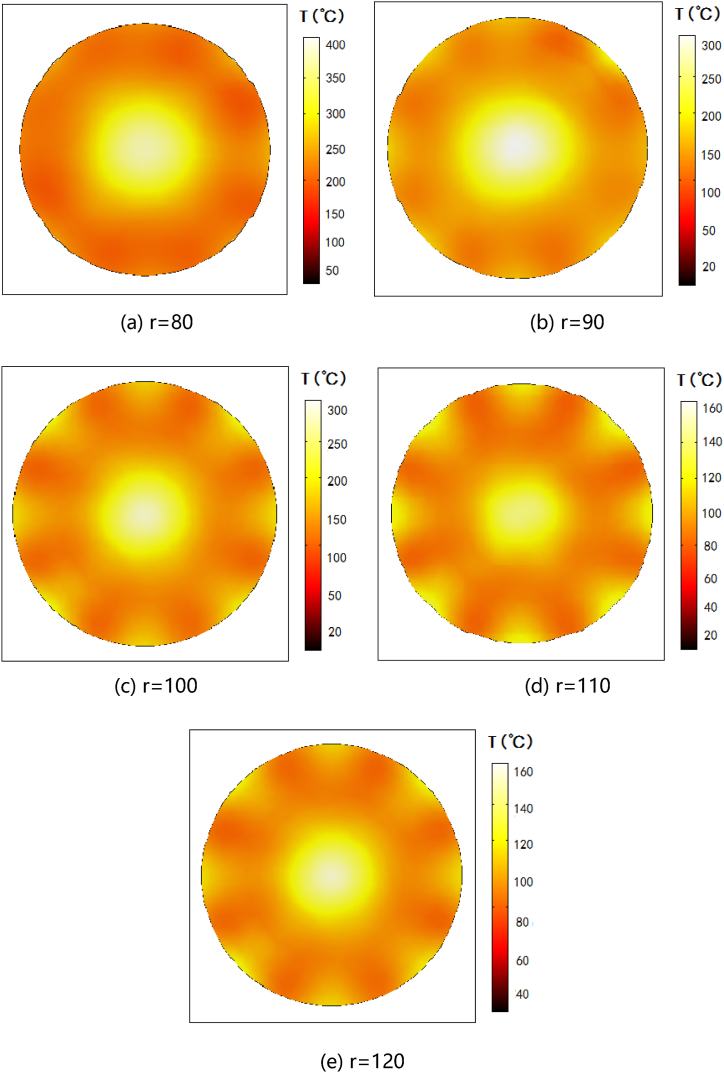
Different pipe radii correspond to microwave heating cross-section temperature distribution.

Through the above analysis, we obtained the optimal design scheme of the waveguide inlets. The main part of the microwave reactor device for MOFs designed in this paper is a pipe for carrying the reaction solution, which the length is based on the number of waveguides distributed around, and the radius of that needs to be further optimized. A large value of the pipe radius will cause non-uniform heating because the skin depth of microwave irradiation of organic solutions is much smaller than the size and scale of industrial devices, as well as microwave losses. However, if the pipe radius is too small to load the horn waveguide, and the processing size is too small, which will lead to a low yield of MOFs and is not convenient for large-scale industrial applications. Therefore, it is necessary to determine the optimal pipe radius to achieve relatively good uniformity of microwave heating. Based on the parameters of the number and distribution of waveguides, the influence of pipeline radius on microwave heating uniformity is discussed in this section. The inner diameter r of the pipe was set as: 80 mm, 90 mm, 100 mm, 110 mm, 120 mm, respectively. The temperature distribution corresponding to microwave action under different radii was obtained through numerical simulation, as shown in Fig. 7.

Observe Fig. 7, as the inner diameter of the pipeline reactor chamber changes, the temperature rise obtained by the reaction chamber changes significantly. When r = 80 mm, the maximum temperature is close to 400 °C, with the gradual increase of r, the temperature gradually decreases, when r = 120 mm, the maximum temperature is only 160 °C. In summary, it shows that the smaller the inner diameter, the less yield of the MOFs synthetic, and the more concentrated the microwave energy range, the higher the temperature rise. Or say, there is a negative correlation between yield and temperature rise. From the color distribution in Fig. 7(a)–(e), it can be seen that the highest temperature rise area is concentrated in the center of the reaction cavity. This is because there are 8 waveguides around the pipeline, and the superposition of electric field will occur at the center of the reaction cavity, so the temperature rise at the center is higher than that in other areas.

**Fig. 8:**
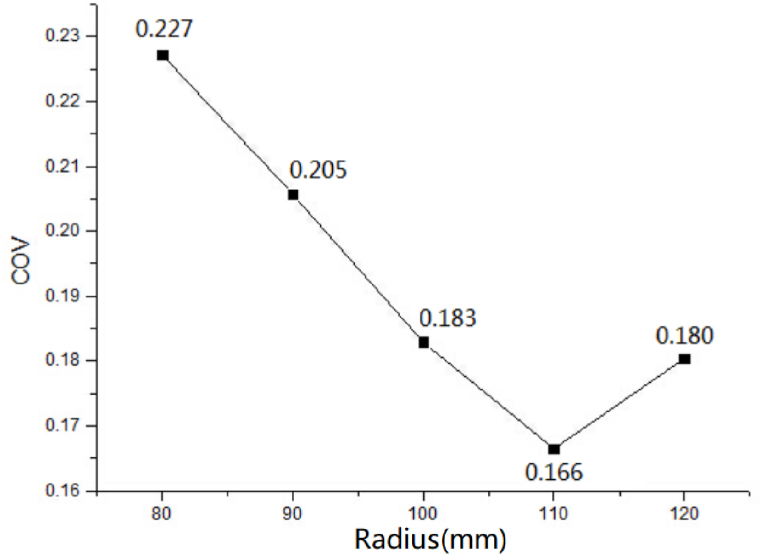
Temperature field COV curve of different pipe radii.

The temperature field distribution about microwave heating is not enough to obtain the optimal pipe radius, and the heating uniformity needs to be further quantified to get more accurate results. Here, the temperature field COV values corresponding to different pipe radii are calculated, and the curves are drawn as Fig. 8.

As the Fig. 8 shows, when the inner diameter r increases from 80 mm to 110 mm, the temperature field COV decreases. While the r continues to increase to 120 mm, the temperature field COV increases. This indicates that the uniformity of the temperature field first increases and then decreases when the inner diameter of the pipeline r increases. We analyzed that why the critical point of 110 mm exists in this model, which is as the inner diameter is small, the superposition of the electric field in the center produces too high temperature rise,and result the poor overall heating uniformity. However, when the inner diameter is over this critical point, although the central temperature rise is reduced, the microwave radiation range of the surrounding waveguide inlet is smaller, resulting in a large temperature difference, and result the overall uniformity becomes worse. To sum up, when the number and distribution parameters of waveguides are n = 8 and α = 45°, the optimal inner diameter r is 110 mm, which can achieve the optimal temperature field uniformity of MOFs synthesis.

Microwave assisted MOFs synthesis has the advantages of fast reaction speed, high yield, controlling crystal size, low energy consumption, little or no chemical waste and so on. Many research teams at home and abroad are committed to expanding the experimental scale of microwave synthesis, but they are faced with key problems such as controllability of material synthesis conditions and thermal stability of microwave reactors. In this study, a pipeline microwave reaction device is proposed for the first time. Based on the finite element method, the geometric structure of the reaction device was optimized by coupling the mathematical model of electromagnetic field, chemical reaction, temperature field, etc., and the heating uniformity was effectively improved. We found that the optimal radius of the tube type reaction device was 110 mm, which was greatly improved from the reaction scale of 100 ml × 4 reported before [29]. At the same time, the microwave utilization rate of the optimized pipeline reactor is about 96.25 %, which is much higher than that of the traditional microwave reactor [30].

### 3.2 Process parameters optimization for microwave synthesis of MOFs by orthogonal method

#### 3.2.1 Orthogonal experimental design of multi-physics simulation

The orthogonal experiment design to optimize the process parameters of MOFs growth by pipeline microwave reactor device includes the following: selection of test targets, evaluation of indicators, selection of factors and levels, design of appropriate orthogonal array, test plans and corresponding results. Based on range analysis, matrix analysis and variance analysis, the test results were analyzed to find the optimal combination of factors and levels.

Based on the finite element method, the density field, pressure field and temperature field of MOFs growth by microwave heating were simulated. Take the simulation results of five points on the heating cross-section, and calculate the variance values of each physical field according to equation (2):

(2) $$S^{2}=\frac{{\sum_{1}^{n}\left(x_{i}-m\right)}^{2}}{n-1}$$

Where, $x_{i}$ represents the simulation value of a sampling point, m is the average value, n is the amount of data, n = 5.

As shown in Table 3, the first column lists numbers from 1 to 16, representing 16 simulation cases. Columns 2 to 4 represent the four levels of three different factors A, B, C, respectively. Considering that the numerical simulation of this study involves three variable factors, the last column is listed as the error evaluation, namely error term.

**Table 3:** L_12_(3^4^) orthogonal designed scheme and results.

Case	A	B	C	Error term	Density variance	Pressure variance	Temperature variance
1	1	11	1	1	4.3111 × 10^−11^	6.1272 × 10^−8^	1.4066 × 10
2	1	2	2	2	1.2756 × 10^−10^	3.2569 × 10^−7^	1.2143 × 10
3	1	3	3	3	3.1201 × 10^−10^	9.1130 × 10^−7^	1.1205 × 10
4	1	4	4	4	4.1559 × 10^−10^	1.7154 × 10^−6^	1.4756 × 10
					0		
5	2	1	2	4	8.1344 × 10^−11^	6.3754 × 10^−7^	1.0231 × 10
6	2	2	1	3	2.5194 × 10^−10^	1.3698 × 10^−6^	0.8750 × 10
					1		
7	2	3	4	2	1.2261 × 10^−10^	8.6731 × 10^−8^	1.5938 × 10
8	2	4	3	1	2.1182 × 10^−10^	1.1245 × 10^−7^	1.2849 × 10
9	3	1	3	2	9.2721 × 10^−11^	1.1273 × 10^−6^	1.0944 × 10
10	3	2	4	1	1.8423 × 10^−10^	7.1791 × 10^−7^	0.8317 × 10
11	3	3	1	4	2.0907 × 10^−10^	3.1233 × 10^−7^	1.2127 × 10
12	3	4	2	3	2.0932 × 10^−10^	1.0182 × 10^−7^	1.1861 × 10
13	4	1	4	3	5.1125 × 10^−11^	2.0215 × 10^−7^	1.4845 × 10
14	4	2	3	4	6.6971 × 10^−11^	5.7988 × 10^−8^	1.6571 × 10
15	4	3	2	1	4.6779 × 10^−10^	1.4609 × 10^−6^	1.3180 × 10
16	4	4	1	2	4.7499 × 10^−10^	6.7650 × 10^−7^	1.0908 × 10

In Table 3, each row in the table corresponds to a growth condition, where the numbers “1–4” represent the different levels of each factor. For example, the 6th case corresponds to a combination of 2 level of A, the 2 level of B, and the 1 level of C. The right columns on the right side of the table list the indicators, namely the density variance, pressure variance and temperature variance for each case.

#### 3.2.2 Range analysis

Range analysis is an effective method to analyze the test results, and the average value of each factor is used as the evaluation standard of the primary and secondary order and the optimal level. The range calculation method is:

1) $\bar{K1}$, $\bar{K2}$, $\bar{K3}$, and $\bar{K4}$ represents the average value of each level. The smaller the average value of the test results, the smaller the variance, and the better the uniformity of the film under this condition.
2) The range R=max{K1,K2,K3,K4}−min{K1,K2,K3,K4} indicates the influence of the corresponding factors at each level of the test results on the test index.

The larger the level, the more important the influence of the level change on the test index. The range of the error term is the error generated randomly. If the range of error terms in a test result is large, it indicates that there is an interaction between the factors. Through the experimental simulation of 12 orthogonal combinations, the primary and secondary order of each factor and the optimal level of temperature distribution, pressure distribution and density distribution are obtained. The range calculation results are shown in Table 4:

**Table 4:** Range distribution and primary and secondary factors of orthogonal test.

index	criteria	Factor			
		A	B	C	Error Term
Density variance	${\bar{K}}_{1}$	1.62 × 10^−11^	2.52 × 10^−10^	2.51 × 10^−10^	2.19 × 10^−10^
	${\bar{K}}_{2}$	6.68 × 10^−10^	2.23 × 10^−10^	1.68 × 10^−10^	2.14 × 10^−10^
	${\bar{K}}_{3}$	2.89 × 10^−10^	1.92 × 10^−10^	1.82 × 10^−10^	2.30 × 10^−10^
	${\bar{K}}_{4}$	3.29 × 10^−10^	3.12 × 10^−10^	2.68 × 10^−10^	1.89 × 10^−10^
	R	2.63 × 10^−10^	1.23 × 10^−10^	1.11 × 10^−10^	3.30 × 10^−11^
	Order	A_1_-B_3_-C_2_			
Pressure variance	${\bar{K}}_{1}$	5.41 × 10^−7^	7.37 × 10^−7^	7.48 × 10^−7^	5.68 × 10^−7^
	${\bar{K}}_{2}$	6.49 × 10^−7^	6.13 × 10^−7^	5.80 × 10^−7^	5.88 × 10^−7^
	${\bar{K}}_{3}$	6.74 × 10^−7^	5.81 × 10^−7^	6.01 × 10^−7^	6.69 × 10^−7^
	${\bar{K}}_{4}$	6.46 × 10^−7^	5.75 × 10^−7^	5.81 × 10^−7^	6.83 × 10^−7^
	R	1.37 × 10^−7^	1.59 × 10^−7^	1.71 × 10^−7^	1.04 × 10^−7^
	Order	C_2_-B_4_-A_1_			
Temperature variance	${\bar{K}}_{1}$	12.96	11.32	12.89	12.39
	${\bar{K}}_{2}$	11.48	12.11	12.69	12.68
	${\bar{K}}_{3}$	13.01	12.78	11.61	12.19
	${\bar{K}}_{4}$	13.60	15.27	13.96	14.06
	R	1.90	4.10	2.37	1.71
	Order	B_1_-C_3_-A_2_			

As the results in the Table 4, the error terms of the three indexes is smaller than that of three factors, indicating that the factors have almost no mutual influence and indicating the rationality of the orthogonal experiment design. For the density field, R_A_(2.63 × 10^−10^) > R_B_(1.23 × 10^−10^) > R_C_(1.11 × 10^−10^), therefore the order of primary and secondary effects of factors is A-B-C. The minimum average $\bar{K}$ of each factor is selected as the optimal value. Therefore, the optimal horizontal combination of the ranking of the primary and secondary factors of density field is: A_1_-B_3_-C_2_. By the same way, the optimal horizontal combination of the primary and secondary factors of pressure field and temperature field is C_2_-B_4_-A_1_ and B_1_-C_3_-A_2_.

#### 3.2.3 Matrix analysis

The range analysis method is used to judge the main and secondary order of each factor and the optimal combination of levels corresponding to different indicators appear different optimization results, and the weight of each factor and level is further analyzed. The matrix analysis model of orthogonal experiment includes index layer, factor layer and horizontal layer. Follow these steps to perform matrix analysis:

Step 1 Establish the M, T, S matrix of each factor and then calculate the weight matrix $k_{i}\left(i=1,\ldots ,m\right)$.

Step 2 Establish the mean value matrix of m matrices, and its mean value can be obtained by $\sum_{j=1}^{m}k_{i}/m$. The k matrix is:

(2) $$k^{T}=\left(\begin{array}{ccccccc} A_{11} & \cdots & A_{1n} & \cdots & A_{m1} & \cdots & A_{mn} \end{array}\right)$$

which $A_{\text{ij}}\left(i=1,\ldots ,m;j=1,\ldots ,n\right)$ is the index value corresponding to the jth level in the ith factor column.

Step 3 Mark the maximum or minimum value corresponding to each factor in the k matrix, and set the optimal factor as the level corresponding to the extreme value j, in order to obtain the optimal level combination of each factor.

According to the above steps, the weight matrices of density field, pressure field and temperature field are $k_{1}$, $k_{2}$, $k_{3}$ respectively:

(3) $$k_{1}=M_{1}T_{1}S_{1}=\left(\begin{array}{l} 0.1202\\ 0.1437\\ 0.1186\\ 0.1245\\ 0.1391\\ 0.1502\\ 0.1255\\ 0.1318\\ 0.1197\\ 0.1523\\ 0.1411\\ 0.1209 \end{array}\right)\;k_{2}=M_{2}T_{2}S_{2}=\left(\begin{array}{l} 0.0081\\ 0.0076\\ 0.0094\\ 0.0080\\ 0.0073\\ 0.0076\\ 0.0084\\ 0.0092\\ 0.0085\\ 0.0072\\ 0.0119\\ 0.0089 \end{array}\right)\;k_{3}=M_{3}T_{3}S_{3}=\left(\begin{array}{l} 0.0045\\ 0.0053\\ 0.0057\\ 0.0039\\ 0.0082\\ 0.0071\\ 0.0046\\ 0.0057\\ 0.0061\\ 0.0048\\ 0.0059\\ 0.0070 \end{array}\right)$$

Finally, the average weight matrix is obtained:

(4) $$k=\frac{k_{1}k_{2}k_{3}}{3}=\left(\begin{array}{l} 0.0442\\ 0.0522\\ 0.0446\\ 0.0455\\ 0.0515\\ 0.0550\\ 0.1385\\ 0.0489\\ 0.0448\\ 0.0548\\ 0.0530\\ 0.0456 \end{array}\right)$$

Through the calculation and analysis of the weight matrix above, the optimal horizontal combination is obtained: A_2_-B_2_-C_2_. This shows that the optimal process parameters of MOFs growth by pipeline microwave reactor device is: microwave power is 200 W, irradiation time is 100 min, and reagent concentration is 50 mM/L.

**Fig. 9:**
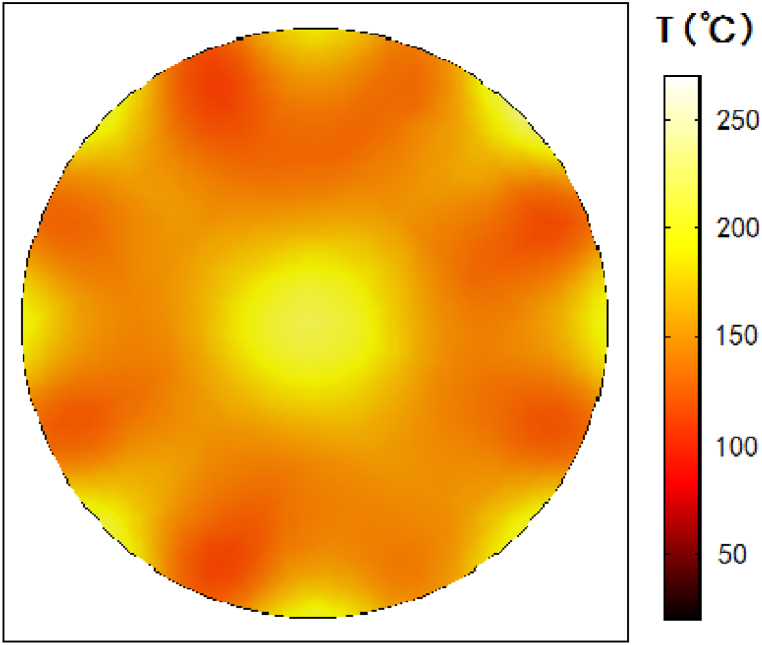
Temperature field distribution in cross section of MOFs synthesized by microwave heating.

Based on the above optimized process parameter combination, the multi-physics simulation calculation of MOFs growth by pipeline microwave reactor device has been carried out. The temperature field distribution of the cross section of the microwave reactor device is shown in the Fig. 9.

Due to the superposition of electric field in the center of the reactor, the temperature of the center is too high. When microwaves radiation to material with electromagnetic losses, the electric field strength decays rapidly, creating a temperature gradient from the edge to the center of the device in the Fig. 9. The coefficient of variation of the temperature field is relatively small as 0.166, that is the degree of dispersion of the temperature field is small, indicating that the process of synthesizing MOFs by microwave heating in this reaction device has good heating uniformity. The microwave power is 200 W, the microwave absorbed by the reactants is about 193.3 W, so the microwave power utilization rate is 96.65 %, which is much higher than the microwave utilization rate of traditional microwave oven as a reactor.

In this part, we use the orthogonal method to analyze the comprehensive error and accuracy of range, matrix and variance of the simulation results of multi-physical field coupling calculation of density field, pressure field and temperature field in the process of microwave assisted method, and discuss the primary and secondary effects of each factor. With regard to the temperature field, the microwave acts as the heat source of the reaction, converting electromagnetic field energy into thermal field energy by radiation. Where electromagnetic wave acts on chemical reaction, its reactants and products will participate in the influence of heat conduction, and the change of energy transfer can be simulated by fractional dynamics in heat transport [23]. At present, because the microwave reaction device is still in the design stage of simulation, after the reaction system is built in the later stage, we will specifically analyze the heat transfer part of microwave assisted synthesis based on fractional calculus method, and submit the corresponding experimental data.

## 4 Conclusion

Based on the application advantages and outstanding material properties of MOFs materials in many fields, it is of great significance to find a synthesis method that is efficient, energy-saving, green and more conducive to industrial promotion. The research work of this paper is to solve the problems of low efficiency, small processing capacity, uneven heating and low reproducibility in the traditional microwave assisted synthesis of MOFs materials. The specific research results are as follows:

1. A pipeline type microwave reaction device is proposed in this study. Based on the numerical calculation of multi-physical fields such as electromagnetic field, heat transfer and chemical reaction, the geometric results of the reaction device are optimized. Waveguide feeder should adopt trumpet structure, the optimal number of waveguide and distribution angle parameters are: n = 8, α = 45°, the optimal pipe radius is 110 mm. The simulation results show that the thermal field is evenly distributed without thermal runaway, and the microwave utilization rate reaches about 96.25 % [29].
2. The key process parameters for synthesis of MOFs materials were optimized by orthogonal experiment and error analysis to obtain the optimal combination suitable for this model: microwave power is 200 W, irradiation time is 100 min, and reagent concentration is 50 mM/L.

As for the design and optimization of the reaction device for the synthesis of MOFs materials by microwave method, although the theoretical analysis is carried out in detail, the comparison with the existing literature reports shows that the simulation results are good and basically meet the design objectives. However, some data are still lacking in experimental verification. Based on the research work in this paper, we will process and build a microwave reaction device suitable for MOFs synthesis in the future, and carry out confirmatory experiments based on simulation data. By comparing experimental data with theoretical data, we can further verify the correctness and feasibility of the design scheme [29]. And provide effective data reference for further optimization.

In summary, this paper proposes a new type of pipeline microwave reaction device, which improves the reaction scale of MOFs material synthesis, improves the microwave utilization rate, and improves the synthesis efficiency and reaction uniformity. It plays an important guiding role to further promote the industrial application of microwave synthesis of MOFs material, and reflects its importance in scientific research and practical application.

## CRediT authorship contribution statement

**Jiadai An:** Writing – review & editing, Writing – original draft, Funding acquisition, Data curation, Conceptualization. **Xianying Dai:** Software, Data curation. **Ying Liu:** Software, Conceptualization. **Kama Huang:** Resources. **Dengke Zhang:** Writing – review & editing.

## Declaration of competing interest

We declare that we have no financial and personal relationships with other people or organizations that can inappropriately influence our work, there is no professional or other personal interest of any nature or kind in any product, service and company that could be construed as influencing the position presented in, or the review of, the manuscript entitled, “Design and process optimization of a new reaction chamber for microwave synthesis of MOFs materials”.
